# Immunogenicity of monkeypox virus surface proteins and cross-reactive antibody responses in vaccinated and infected individuals: implications for vaccine and therapeutic development

**DOI:** 10.1186/s40249-025-01280-1

**Published:** 2025-02-25

**Authors:** Jing Liu, Xun Wang, Yiting Zhang, Changyi Liu, Meng Zhang, Chen Li, Peiling Liu, Shanshan Li, Kaifeng Wei, Yiming Cai, Hongjie Yu, Zhiliang Hu, Pengfei Wang, Yanliang Zhang

**Affiliations:** 1 Department of Infectious Diseases, Nanjing Research Center for Infectious Diseases of Integrated Traditional Chinese and Western Medicine, Nanjing Hospital of Chinese Medicine Affiliated to Nanjing University of Chinese Medicine, Nanjing, Jiangsu China; 2 Shanghai Pudong Hospital, Fudan University Pudong Medical Center, State Key Laboratory of Genetic Engineering, MOE Engineering Research Center of Gene Technology, School of Life Sciences, Shanghai Institute of Infectious Disease and Biosecurity, Shanghai Key Laboratory of Oncology Target Discovery and Antibody Drug Development, Fudan University, Shanghai, China; 3 Department of Infectious Disease, The Second Hospital of Nanjing, Nanjing University of Chinese Medicine, Nanjing, Jiangsu China; 4 Shanghai Institute of Infectious Disease and Biosecurity, Fudan University, Shanghai, China; 5 Nanjing University of Chinese Medicine, Nanjing, Jiangsu China; 6 Center for Global Health, School of Public Health, Nanjing Medical University, Nanjing, Jiangsu China

**Keywords:** Monkeypox, Cross-reactive antibody response, Immunogenicity, Entry fusion complex

## Abstract

**Background:**

The monkeypox virus (MPXV) has raised global health concerns due to its widespread transmission. This study evaluated the MPXV immunogenic antigens and the impact of vaccinia virus (VACV) vaccination and MPXV infection on cross-reactive antibody responses to conserved proteins from representative MPXV strains that reflected the evolutionary trajectory.

**Methods:**

Phylogenetic analyses were first conducted to reveal the evolutionary trajectory of MPXV from 1970 to 2024. A total of 84 serum samples were collected: 42 from VACV-vaccinated individuals, 12 from MPXV-infected participants in the early stage, 13 from the late stage, and 17 from naive individuals. Demographic data, MPXV and HIV status, as well as other clinical information were collected using standardized forms. Immunogenicity, cross-reactive antibody responses, and amino acid similarity to 15 MPXV surface proteins were assessed using enzyme-linked immunosorbent assays, VACV neutralization tests, and sequence alignment. Data analysis methods included analysis of variance, Mann–Whitney U test, binary logistic regression, Pearson correlation, and linear regression, with a significance threshold of *P* < 0.05.

**Results:**

The 186 complete genome sequences were classified into different clades and lineages, ranging from clade Ia to clade IIb C.1.1. Individuals infected with MPXV demonstrated strong antibody responses to antigens A35R, B6R, H3L, and E8L. VACV-vaccinated individuals exhibited broader cross-reactivity, particularly against A21L (*P* = 0.0003), A28L (*P* = 0.0028), A29L (*P* = 0.0324), G2R (*P* = 0.0003), and H2R (*P* = 0.0008), compared to MPXV-infected individuals. Pearson correlation analysis revealed significant associations (*P* = 0.0049) between antibody responses and the amino acid sequence similarity with other orthopoxviruses. Furthermore, MPXV-infected individuals exhibited greater neutralizing activity against VACV than those VACV-vaccinated individuals (*P* < 0.0001), while the vaccinated group retained cross-protective immunity even decades post-vaccination.

**Conclusions:**

A35R, B6R, H3L, and E8L are the main immunogenic antigens of MPXV. VACV-vaccination triggers a cross-reactive antibody response to MPXV surface proteins. Our findings suggest the need for targeted vaccines and antibody treatments for MPXV, as well as the reintroduction of smallpox vaccinations with booster doses for high-risk groups.

**Graphical Abstract:**

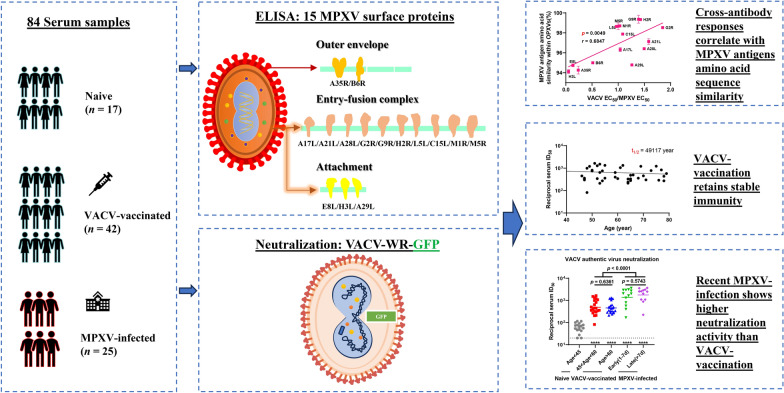

**Supplementary Information:**

The online version contains supplementary material available at 10.1186/s40249-025-01280-1.

## Background

The monkeypox virus (MPXV), an orthopoxvirus (OPXV), has spread to 112 countries and regions since May 2022 [1]. The World Health Organization (WHO) declared monkeypox (mpox) a Public Health Emergency of International Concern on July 23, 2022, and the first imported case in Chinese mainland was reported on September 16, 2022 [2]. As of November 30, 2024, 117,633 confirmed cases and 2 suspected cases including 263 deaths have been reported to WHO [3]. Furthermore, owing to the increase in mpox cases in the Democratic Republic of the Congo (DRC) and other African countries, the WHO renewed the Public Health Emergency of International Concern on August 14, 2024 [4]. The ongoing mpox outbreak in the DRC is linked to a new and more lethal sublineage of MPXV clade I, which differs from previously circulating strains, and has seen an increased in prevalence among females and adolescents [5–7]. On November 15, 2024, California reported its first case of clade I mpox [8]. In light of the recent MPXV clade Ib cases has been reported in China [9], it is imperative to monitor genetic and amino acid alterations in the MPXV. Such changes may present considerable obstacles to the swift response, management, and elimination of mpox outbreaks.

The highly conserved amino acid sequences of OPXVs enable immunogenicity and immune response studies using serum samples from current mpox patients and individuals previously vaccinated against smallpox [10]. The Tiantan strain of vaccinia virus (VACV) was utilized to protect millions of people in China from smallpox until the WHO declared that the world was free of naturally occurring smallpox in 1980 [11]. Research suggests that antibodies induced by smallpox vaccination may provide cross-protection against MPXV, helping to shield the population from infection [12, 13]. Studies have shown that individuals vaccinated against VACV display cross-reactivity to certain MPXV surface proteins, including H3L, H8L, A35R, B6R, A29L, and M1R [14–17]. However, current research on MPXV often focuses on a limited range of functional proteins, and a thorough evaluation of the antigen–antibody response is lacking.

OPXVs, including MPXV and VACV, contain large linear double-stranded DNAs that encode up to 200 viral proteins. Among these proteins, entry-fusion proteins are conserved across all poxviruses, and form a complex known as the entry fusion complex (EFC) [18]. The infectious virions of MPXV exist in two forms: the intracellular mature virion (IMV) and the extracellular enveloped virion (EEV). MPXV specifically uses four proteins for attachment: A29L, H3L, E8L, and A28L [19]. The surface proteins of mature virion (MV), such as H3L, D8 (E8L in MPXV), A27L (A29L in MPXV), L1(M1R in MPXV), A28L and A17L, as well as those of enveloped virions (EVs), such as B5 (B6R in MPXV) and A33 (A35R in MPXV), are recognized as targets for neutralizing antibodies, highlighting the complementary roles of MVs and enveloped particles [20].

Therefore, it is crucial to carefully verify and analyze the cross-reactive antibody response and immunogenicity induced by MPXV infection or VACV vaccination, alongside control groups of individuals who have not been infected with MPXV or vaccinated with VACV. In this research, we performed MPXV antigen ELISAs and VACV neutralization tests, highlighting the importance of smallpox vaccination and the generation of cross-reactive antibody responses to MPXV from various previous OPXV exposures, which offers insights for future vaccination and treatment strategies.

## Methods

### Evolutionary tree analysis for the evolutionary trajectory and representative strains of MPXV

This evolutionary trajectory is to demonstrate the evolutionary trajectory and key events of MPXV from 1970 to 2024. The genomic sequences of Monkeypox from 1970 to 2024 were downloaded from NCBI (https://www.ncbi.nlm.nih.gov/nuccore/?term=monkeypox#) (accessed August 28, 2024) and National Genomics Data Center (https://ngdc.cncb.ac.cn/genbase) (accessed August 28, 2024). KJ642613.1 (9-month-old child, the first recognized human mpox case in medical history) and DQ011156.1, both identified in 1970, served as references for MPXV clade Ia and MPXV clade IIa, respectively [21]. A total of 186 complete genome sequences were uploaded into MAFFT v7.703b to complete the alignment and the result was imported into MEGA 11.0 to conduct Neighbor-Joining tree (Bootstrap 500) [22, 23]. Analyses were conducted via the Maximum Composite Likelihood model [24]. The visualization of the evolutionary tree was accomplished by Interactive Tree of Life (iTOL) v6 (biobyte solutions GmbH, Heidelberg, Germany) [25]. From this assay, the representative MPXV strains (from clade Ia to clade IIb C.1.1) could be selected to analyze amino acid similarity,

### Cohort design and collection of samples

This study was conducted on serum samples from people who had never been vaccinated with VACV-Tiantan (born after 1980,* n* = 17), people who had been vaccinated with VACV-Tiantan (born before 1980, *n* = 42), and MPXV-infected individuals (11 patients provided samples at both the early and late stages of infection, while 3 patients only participated in one-time sample collection. sample *n* = 25 and individual *n* = 14). The participants in this trial were enrolled and their blood samples were collected from December 2023 to June 2024 in Nanjing Hospital affiliated to Nanjing University of Chinese Medicine (the Second Hospital of Nanjing) and Nanjing Hospital of Chinese Medicine affiliated to Nanjing University of Chinese Medicine. We collected the sex and age information of all the participants. For the blood samples of those infected with MPXV, we additionally conducted tests for monkeypox viral load, human immunodeficiency virus (HIV) viral load, T cell count, and routine blood examination (antiretroviral therapy beginning time, white blood, neutrophil, lymphocyte, blood platelet, hemoglobin, CD4 + T cell count, CD8 + T cell count, HIV viral load, and MPXV CT value) for further factor correlation analysis. Monkeypox virus nucleic acid detection kit (Real-Time Quantitative Reverse Transcription) (Da'an Gene, Guangzhou, China) was utilized to conduct MPXV CT value test. Serum samples were isolated by centrifugation of coagulated whole blood, and aliquoted for storage at − 80 °C. Roche Cobas AmpliPrep/Cobas TaqMan HIV-1 Test, version 2.0 (F. Hoffmann-La Roche Ltd, Basel, Switzerland) was utilized to perform the HIV viral load test.

### Immunogenicity and cross-reactive antibody responses

#### Expression and purification of 15 MPXV protein antigens

Recombinant MPXV protein antigens were constructed, expressed and purified for use in the enzyme-linked immunosorbent assay (ELISA). The nucleotide sequences for the extracellular domain of A17L (residues 1 to 341), A21L (residues 22 to 115), A28L (residues 22 to 146), A29L (residues 21 to 110), A35R (residues 58 to 181), B6R (residues 18 to 279), C15L (residues 1 to 175), E8L (residues 1 to 275), G2R (residues 22 to 111), G9R (residues 1 to 319), H2R (residues 50 to 189), H3L (residues 1 to 278), L5L (residues 1 to 111), M1R (residues 3 to 183), and M5R (residues 52 to 128) from MPXV (2022 MPXV, GenBank accession numbers: PP778666.1) were synthesized (Huajin, Shanghai, China) and inserted into the mammalian expression vector pCMV3 (8 × His tag). The Expi293F cells were then cultured at 37 °C, shaking at 125 rpm with 8% CO_2_ for 5 days. The supernatant was collected after centrifugation at 4000×*g *for 10 min and Ni-Smart beads (Smart-Lifesciences, Shanghai, China) were added and incubated for 1 h at room temperature. The protein was purified with imidazole and then transferred to PBS via the 10 kDa Amicon^®^ Ultra centrifugal filter (Millipore, Darmstadt, Germany) and evaluated the purity.

#### Single antigen ELISAs

Research has indicated that the surface proteins of VACV are capable of eliciting neutralizing antibodies that possess both prophylactic and therapeutic efficacy, as well as their corresponding homologs in MPXV. We established the serologic ELISA to measure the antibody responses of individuals and immunogenic antigens. ELISAs utilizing 15 purified recombinant MPXV antigens from the above were all performed via the same method, with variation in the coating antigen only. Briefly, the high-binding 96-well plate was coated with 100 µL/well of 2 µg/mL recombinant antigen overnight at 4 °C. After overnight incubation, plates were blocked by with 100 μL blocking buffer (3% BSA in PBS) at 37 °C for two hours. The samples were serially diluted (from 1∶50 folding with fivefold-serial dilutions) using dilution buffer (1% BSA in PBS), incubated at 37 °C for one hour. 100 μL of 250-fold diluted goat anti-human IgG (H + L) antibody (Beyotime, Shanghai, China) was added into each well and incubated for 1 h at 37 °C. The plates were washed three times between each step with PBST (0.5% Tween-20 in PBS). The TMB substrate was subsequently added and incubated until the reaction was terminated with terminating reagent (Beyotime, Shanghai, China). The OD_450_ values got from Synergy™ 2 multi-mode microplate reader (Bio Tek, Santa Clara, USA) standardized by subtracting the average of the negative control wells and then dividing by the average maximum signal for each unique coating protein in each experiment.

#### Similarity in representative strain sequence alignments of MPXV surface proteins

An examination of the conservation of MPXV surface proteins across various representative strains is essential for conducting a comprehensive analysis of the conserved proteins contributing to antigenic immune responses in diverse individuals. GenBank accession number of MPXVs (PP778666.1(clade IIb; lineage C.1.1), OR785044.1 (clade IIb; lineage C.1.1), NC_063383.1 (clade IIb; lineage A), ON674051.1 (clade IIb; lineage A.2), PP852946.1 (clade IIb; lineage A.2.3), PP002088.1 (clade IIb; lineage B.1), NC_003310.1 (clade Ia), PP601218.1 (clade Ib), LC831698.1 (clade Ib; lineage C.1)) and aligned VACV (JX489138.1_Tiantan clone TT11, NC_006998.1_Vaccinia virus), cowpox virus (CPXV) (NC_003663.2_Cowpox virus) and smallpox virus (SPXV) (DQ437582.1_Variola virus strain China Horn 1948) sequences were listed in amino acid identity. Amino acid sequences and their homologs by genome annotation in GenBank were aligned and pairwise compared by Jalview software [26, 27].

#### Neutralization assay using VACV

Both MPXV and VACV belong to the OPXV and share over 96% homology in their genome’s central region [28]. Owing to the lack of the BSL-3 laboratory, we used the live VACV strain to evaluate the neutralization ability of the serum samples. 2 × 10^4^ BHK 21 cells were seeded per well in 96-well plate. The VACV-Western Reserve (WR) -GFP recombinant virus (Vector Builder) was incubated from 1∶50 folding with fivefold-serial dilutions of the serum samples for 30 min at 37 °C. The mixtures were subsequently added to the plate and cultivated for another 24 h. The GFP signal was determined via the Operetta CLS, high content analysis system (Perkin Elmer, Shanghai, China). Experimental neutralization titers reported as the serum dilution required to achieve 50% neutralization [29, 30]. The 50% inhibitory dilution (ID_50_) for neutralization, which denotes the dilution factor applied to serum containing antibodies that results in a 50% reduction in in vitro neutralization, is defined as the dilution at which the relative fluorescent units are inhibited by 50% in comparison to the virus control (virus + cells), following the subtraction of the background from the blank control groups (cells only). The ID_50_ values were calculated in GraphPad Prism (GraphPad Software, Boston, USA). The experiment with VACV was performed under BSL-2 conditions.

### Statistical analysis

Continuous variables with normal distribution were presents as mean ± standard deviation; non-normal variables were reported as median (interquartile range, IQR) GraphPad Prism (GraphPad Software, Boston, USA) was utilized for statistical analyses of the virus neutralization and antigen–antibody reaction evaluations as previously reported [31]. Odd ratio (*OR*) analysis of clinical data was performed using SPSS 21.0 (SPSS Inc., Chicago, USA). Multiple-group comparison was done with ANOVA. Mann Whitney test was used to compare the differences in the experiments. Pearson correlation analysis and binary logistic regression analysis were to calculate *OR* and relationships between factors and groups. Linear regression was utilized to describe the trend of the data. *P* values less than 0.05 were considered statistically significant.

## Results

### Evolutionary trajectory and significant events of MPXV from 1970 to 2024

**Fig. 1:**
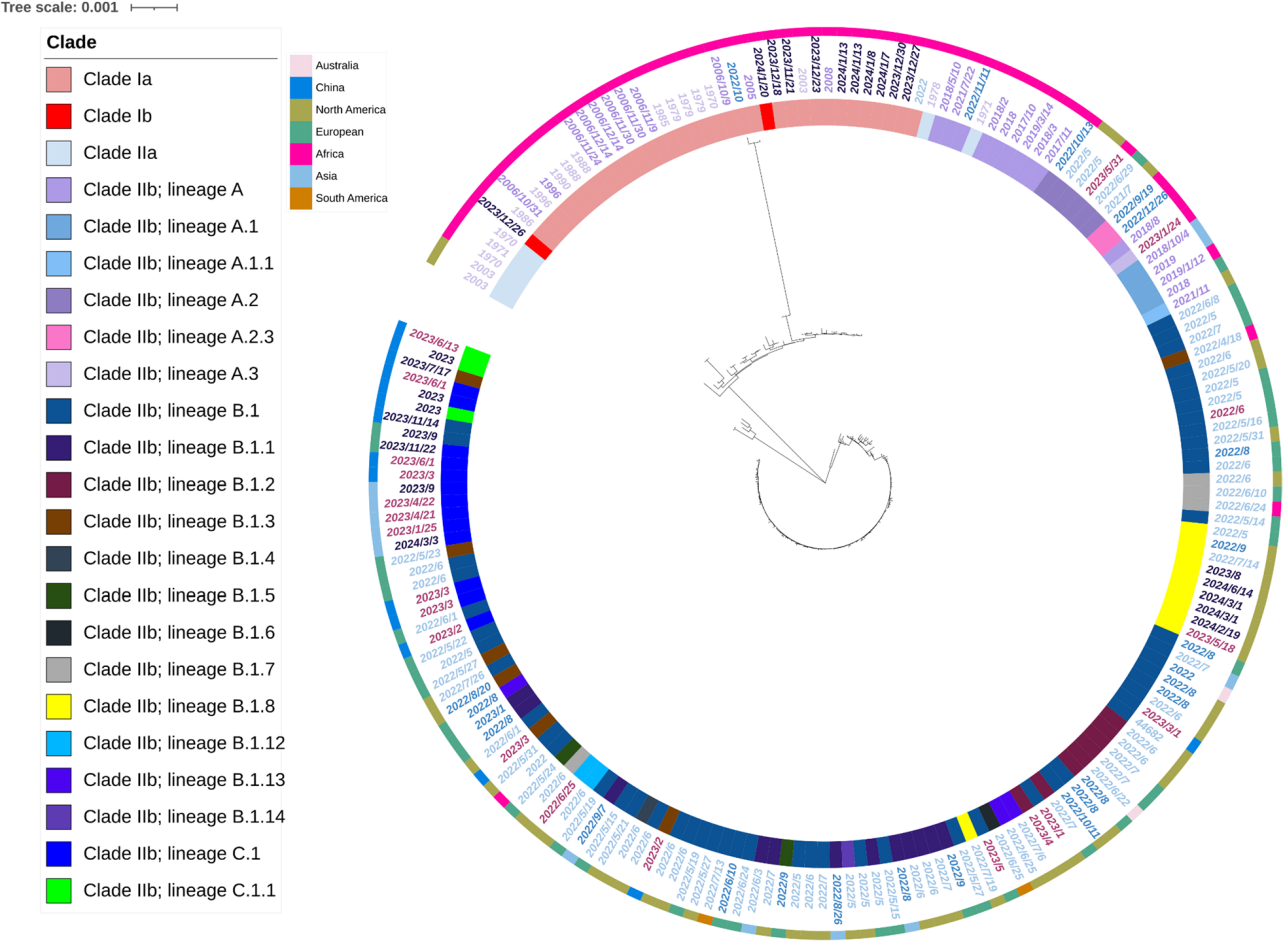
The Neighbor-Joining phylogenomic tree was built using 186 worldwide complete genomes of MPXV, isolated exclusively from human cases between 1970 and 2024, available from NCBI and National Genomics Data Center. The inner color strip represented the clade of sequence labeled by different colors as indicated in the legend in the upper left corner (ranging from pink for clade Ia to bright green for clade IIb lineage C.1.1). The middle annotation ring illustrated the collection date of each case. And the external color strip annotated the continent where each case originated, with cases located in China marked as dark sky blue. The complete information including sequence full name, accession number, location, collection date, and the references for strains could be found in Table S1. MPXV, Monkeypox virus

The original evolutionary trajectory of clade I was evident in our phylogenomic tree, which was marked by red and pink with relatively long and close branches (Fig. 1). They are obviously independent of clade II. In contrast, the 2003 mpox outbreak in the USA, which resulted in fewer fatalities and a lower incidence among patients under 18, was caused by the West African strain (clade IIa), which was imported into the country [32]. A relatively close connection between clades Ia and IIa in our phylogenomic tree indicated the initially slow mutation rate of MPXV. The newly emerged clade Ib, more closely related to the 2003 and earlier variants, exhibited the same characteristics as transmission within adolescents and women [5]. Variants of MPXV arising after 2017 represent a novel lineage potentially linked to clade IIa from Nigeria in the 1970s. The most recent MPXV outbreak, which began in 2022, exhibited epidemiological traits of extensive and rapid transmission, as well as accelerated viral evolution, in contrast to previous outbreaks that were largely confined to a limited number of countries [33, 34]. As of 2024, the global epidemic associated with clade IIb B.1 has significantly diminished. The evolutionary trajectory of clade IIb has undergone further refinement, resulting in the emergence of lineages such as C.1.1, particularly during the latter half of 2023 and into 2024, as illustrated in our phylogenetic tree [35, 36]. This divergent branch may indicate a rapid evolutionary process within clade IIb. An examination of the phylogenetic evolution of MPXV in China revealed that lineages B and C of clade IIb were particularly prevalent in both China and Asia, especially during the period from 2023 to 2024. These strains exhibited a close genetic relationship with variants circulating in certain regions of Europe, implying that the origin of this outbreak was likely imported. Based on these observations, the MPXV clade identified in our study may be classified as clade IIb, lineage C. MPXV from clade Ia to clade IIb; lineage C.1.1 can reflect the variation of MPXV and the changes of antigenic proteins.

### Characteristics of the participants and serum samples in our cohort

Among these MPXV-infected individuals, all 14 were male, with a median age at initial sample collection of 37.5 years (IQR: 32.5–42.0), and 12 of them were tested HIV positive (Table 1). The median duration of known HIV infection among these 12 patients was 3.30 years (IQR: 0.90–5.90). At admission for mpox, the median CD4 + count was 492.50 cells/µl (IQR: 154.50–636.25), and the CD4 + /CD8 + ratio was 0.32 (IQR: 0.22–0.64). Among the 12 patients, 10 had initiated antiretroviral therapy prior to admission, with a median treatment duration of 1.85 years (IQR: 0.08–5.50). Among them, 7 patients had HIV RNA levels below the limit of quantification (20 copies/ml). Ten patients among them had received HIV antiretroviral treatment before the diagnosis of mpox. Additional details regarding the MPXV-infected individuals and samples during the infection phases could be seen in Figure S1. Data pertaining to white blood cell counts, neutrophils, lymphocytes, blood platelets, hemoglobin levels, CD4 + T cell counts, CD8 + T cell counts, HIV viral load, and MPXV CT values were gathered and analyzed in individuals co-infected with MPXV and HIV (Figure S1). A small number of individuals showed CD4 + T cell counts below the normative range, whereas the CD8 + T cell counts of all MPXV-infected subjects remained within the normal range and stabilized over time. The MPXV CT value increased over time and eventually became negative (CT value > 40). Samples collected during the phase ranged from 1 day post infection to 153 days post infection, allowing our research to examine both the short-term and long-term effects of MPXV infection. Most MPXV-infected individuals who were HIV positive had started antiretroviral therapy before their mpox diagnosis, keeping their HIV viral loads under control, except for three individuals; one of these individuals (CD4 + T cell count = 75 cells/μl) took 30 days to test negative for MPXV. All individuals from the naive groups were under 34 years old at the time of sample collection (born after 1989), indicating that they were surely without VACV vaccination protection. In contrast, all individuals in the VACV-vaccinated group were over 47 years old at the time of sample collection (born before 1967), confirming their vaccination status. The sex ratio was fairly even in both groups.

**Table 1:** Baseline characteristics (grouping number, HIV status, gender and age) of enrolled individuals and related serum samples

Baseline characteristics of enrolled individuals				
Variable	Overall	Naive	VACV-vaccinated	MPXV-infected
*n*	73	17	42	14
Age at sample collection, *year*	53 (32–64.75)	30.5 (28–31.25)	59.5 (52–67.5)	37.5 (32.4–42.0)
HIV positive, *n* (%)	12	N/A	N/A	12
Sex, *n* (%)				
Male	47 (64.38%)	9 (52.94%)	24 (57.14%)	14 (100.00%)
Female	26 (35.62%)	8 (47.06%)	18 (42.86%)	0 (0.00%)
Age range, *n*				
< 45	29	17	0	12
45–60	24	0	22	2
> 60	20	0	20	0

Baseline characteristics of enrolled serum samples					
Variable	Overall	Naive	VACV vaccinated	MPXV infected in early infection stage^a^	MPXV infected in late infection stage^b^
*n*	**84**	17	42	**13**	**12**
HIV positive, *n* (%)	**23**	**N/A**	**N/A**	**12 (92.31%)**	**11 (91.67%)**

^a^Samples from some of the 14 MPXV patient and collected according to patients' wishes through infection stage (1~7 days post infection)

^b^Samples from some of the 14 MPXV patient and collected according to patients' wishes through infection stage and late follow-up (> 7 days post infection)

*HIV* human immunodeficiency virus, *MPXV* Monkeypox virus, *VACV* Vaccinia virus

### Serological responses in MPXV-infected and VACV-vaccinated individuals

**Fig. 2:**
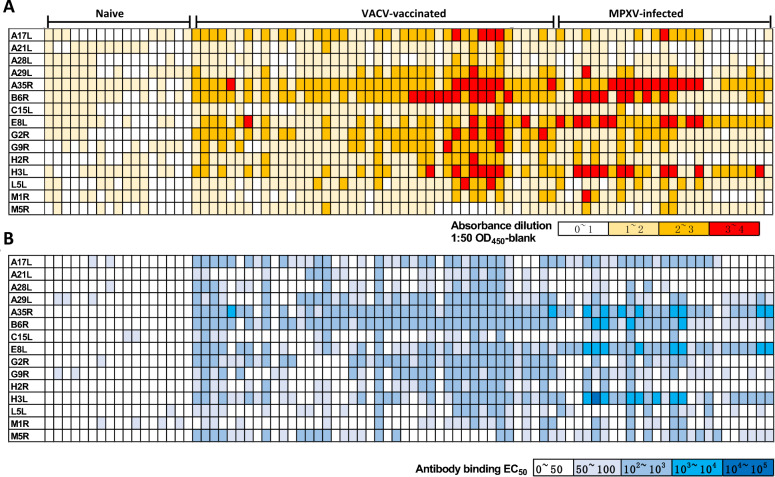
Heatmap of ELISA results using individual MPXV antigens with serum samples from naive (born after 1980 without VACV vaccination and without MPXV infection), VACV-vaccinated without MPXV-infection, and MPXV-infected individuals. **A** Absolute values of the participants’ serum samples binding to the MPXV antigens at a minimum dilution of 1 to 50. **B** Heatmap of the EC_50_ values of the participants’ serum samples binding to the 15 MPXV antigens. The color scale represents the OD_450_ and EC_50_ values. *n* is equal to the number of biologically independent serum samples. The results of individual tests are presented in Figure S2. EC_50,_ Median effect concentration; ELISA, Enzyme-linked immunosorbent assay; MPXV, Monkeypox virus; OPXV, Orthopoxvirus; OD_450,_ Optical density 450; VACV, Vaccinia virus

Absolute optical density 450 (OD_450_) values reflecting the immunogenicity of MPXV antigens across different groups are shown in the heatmap at an initial sample dilution of 1:50 (Fig. 2A). Naive individuals demonstrated minimal antibody binding to all the MPXV antigens. In contrast, individuals who had received smallpox VACV vaccination or had MPXV infection exhibited significantly greater antibody binding to MPXV antigens, with B6R, A35R, E8L, and H3L being the immunodominant antigens (compared with the mean absolute OD_450_ values of other antigens within VACV-vaccinated or MPXV infected samples, all *P* < 0.05). E8L showed variability among individuals, with all MPXV-infected individuals demonstrating robust antibody binding, whereas VACV-vaccinated individuals generally had lower antibody binding (*P* < 0.0001).

Further analysis of antibody binding, indicated by median effect concentration (EC_50_) determination, is illustrated in Fig. 2B. Both VACV-vaccinated and MPXV-infected individuals mounted a highly shared but distinct serological reactivity, significantly surpassing that of naive individuals (*P* < 0.0001). Serum samples from VACV-vaccinated or MPXV-infected individuals continued to exhibit strong antibody binding to B6R, A35R, E8L and H3L in the EC_50_ determination. Notably, serum samples from the VACV-vaccinated group also showed mediocre antibody binding to EFC proteins A17L, G2R, and G9R. In contrast, naive individuals were found to have very weak antibody responses, with most EC_50_ values being 50. VACV-vaccinated individuals without MPXV infection displayed effective antibody responses with EC_50_ values ranging form 10^2^ to 10^3^, while some serum samples from MPXV-infected individuals exhibited excellent antibody binding to B6R, A35R, E8L, and H3L with EC_50_ values ranging form 10^3^ to 10^5^. Multiple individuals have shown simultaneous high levels of antibodies against A35R, B6R, E8L, and H3L. Nonetheless, the antibody binding to A21L and A28L was significantly lower in samples from MPXV-infected individuals compared to those from VACV-vaccinated individuals, although this difference was not apparent in the OD_450_ absolute values. Individuals vaccinated with VACV had robust antibody responses to G2R, G9R, and H2R.

### Differential antibody responses to MPXV surface proteins among VACV-vaccinated, MPXV-infected, and naive individuals

**Fig. 3:**
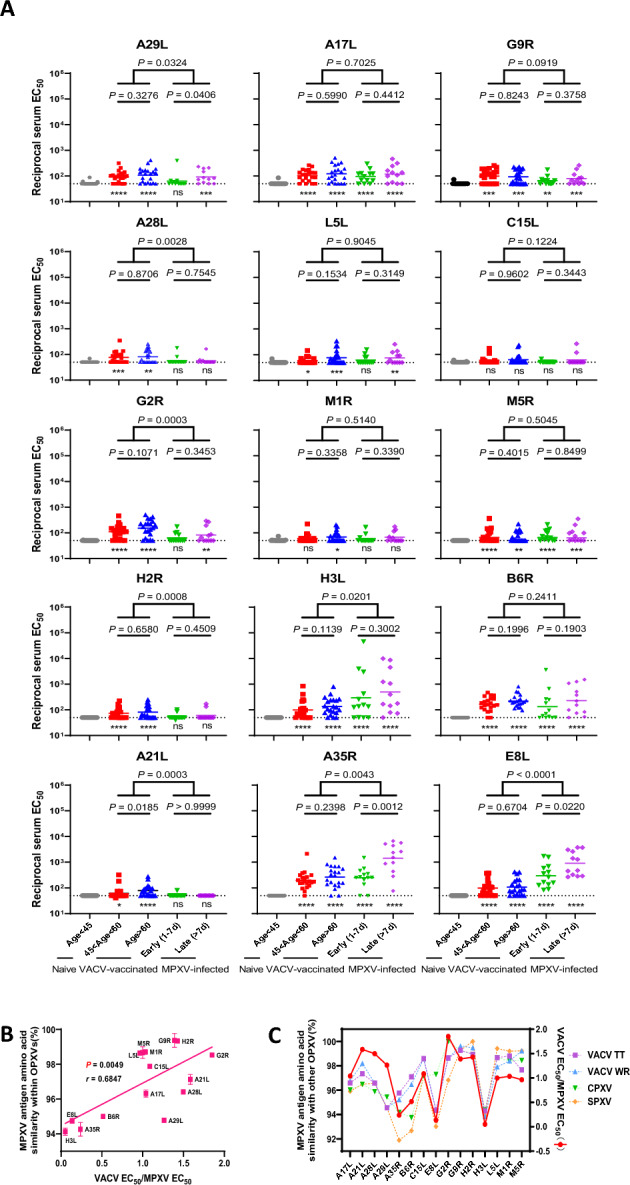
Differential antibody responses to MPXV surface proteins among VACV-vaccinated, MPXV-infected, and naive individuals. **A** Comparison of antibody binding EC_50_ values to distinct MPXV antigens among naive (age < 45), VACV-vaccinated (45 < age < 60 or age > 60) and MPXV-infected (1 ~ 7 or > 7 d.p.i) individuals. **B** Correlation between VACV EC_50_/MPXV EC_50_ values and the amino acid sequence similarity of MPXV proteins with other OPXVs. The sequence similarity is based on the average values obtained by comparing nine MPXV sequences with their homologs in VACV_TT11_001, VACV_WR, Cowpox_Brighton_Red and Variola_virus_strain_China_Horn_1948. **C** The superimposed scatter plots of MPXV antigen amino acid sequence similarity with different OPXVs (VACV TT, VACV WR, CPXV and SPXV) and the VACV EC_50_/MPXV EC_50_ fold change plots. CPXV, Cowpox virus; EC_50,_ Median effect concentration; MPXV, Monkeypox virus; OPXV, Orthopoxvirus; SPXV, Smallpox virus; TT, Tiantan; VACV, Vaccinia virus; WR, Western Reserve

The MPXV-infected samples were further divided according to their collection time, with samples collected at later time point (> 7 days post-infection (d.p.i)) showing overall higher binding EC_50_ values than those collected at earlier time points (1~7 d.p.i), with significant differences for A29L (*P* = 0.0406), A35R (*P* = 0.0012) and E8L (*P* = 0.0220) proteins (Fig. 3A). For the VACV-vaccinated cohort, we categorized the samples by age: individuals over 60 years and those aged between 45 and 60 years. We found that individuals over 60 years of age exhibited similar or even greater (in the case of A21L, *P* = 0.0185) binding EC_50_ values than those aged between 45 and 60 years did, indicating that individuals vaccinated with VACV more than 40 years ago retain antibody cross-reactivity against MPXV surface proteins. When we compared the VACV-vaccinated and MPXV-infected groups, we observed distinct patterns of preferential binding to the proteins in these two groups. Individuals infected with MPXV demonstrated markedly elevated antibody responses to antigens including A35R, E8L, and H3L in comparison to those who had been vaccinated with VACV (*P* < 0.05). Conversely, VACV-vaccinated individuals demonstrated significantly greater binding EC_50_ values for more antigens, such as A21L, A28L, A29L, G2R, and H2R (*P* < 0.05), with some (A21L, A28L and H2R, compared with naive individuals, *P* > 0.05) showing minimal antibody responses in MPXV -infected individuals

We subsequently conducted binary logistic regression analysis to calculate *OR* for factors associated with MXPV antigen recognition (Table S2). The factors assessed included sex, MPXV infection status, VACV-vaccination and age (> 60 years old). The analysis revealed that only antibody levels against G2R were associated with older age, whereas the levels of antibodies against A17L, A29L, B6R, H3L, G9R, G2R and H2R were significantly elevated with VACV-vaccination (*P* < 0.05). Among the MPXV-infected individuals, the CD4+count, CD4+/CD8+ ratio, MPXV CT value and HIV viral load did not affect the antibody response to any antigens (Table S3).

Amino acid similarities were calculated for all 15 MPXV proteins by comparing 9 MPXV strains with VACV Tiantan (TT) (which was used in the SPXV eradication vaccine in China), VACV WR, CPXV, and SPXV (Figure S3 and Table S4). Correlation between amino acid similarity of MPXV antigens within OPXVs and the ratio of antibody binding EC_50_ between VACV-vaccinated individuals and MPXV-infected individuals (VACV EC_50_/MPXV EC_50_) is shown in Figure 3B. The Pearson correlation coefficient was 0.6847 with a *P* value of 0.0049, indicating a significant correlation between amino acid similarity and antibody response in VACV-vaccinated individuals against MPXV-antigens. When we superimposed the scatter plots of MPXV antigen amino acid sequence similarity with different OPXVs and the VACV EC_50_/MPXV EC_50_ fold change plots, we observed that they displayed consistent trends (Figure 3C). VACV-vaccinated individuals demonstrated the most significantly higher binding EC_50_ values against G2R compared to MPXV-infected individuals (*P* = 0.0003) and the highest VACV EC_50_/MPXV EC_50_ (value = 1.85), with G2R showing 98.530% similarity with homologs of OPXVs. In contrast, VACV-vaccinated individuals demonstrated significantly lower binding EC_50_ values against A35R, H3L and E8L compared to MPXV-infected individuals (*P* < 0.05), with their corresponding VACV EC_50_/MPXV EC_50_ values all below 0.237. A35R, H3L and E8L revealed only 94.263%, 94.116% and 94.747% similarity, respectively, with analogues of OPXVs, which were significantly lower than G2R (*P* < 0.05). This suggests that the stronger antibody responses to certain MPXV antigens in VACV-vaccinated individuals are related to the higher amino acid similarity with OPXVs.

### Correlation analysis of antibody binding to MPXV antigens and neutralization against VACV

**Fig. 4:**
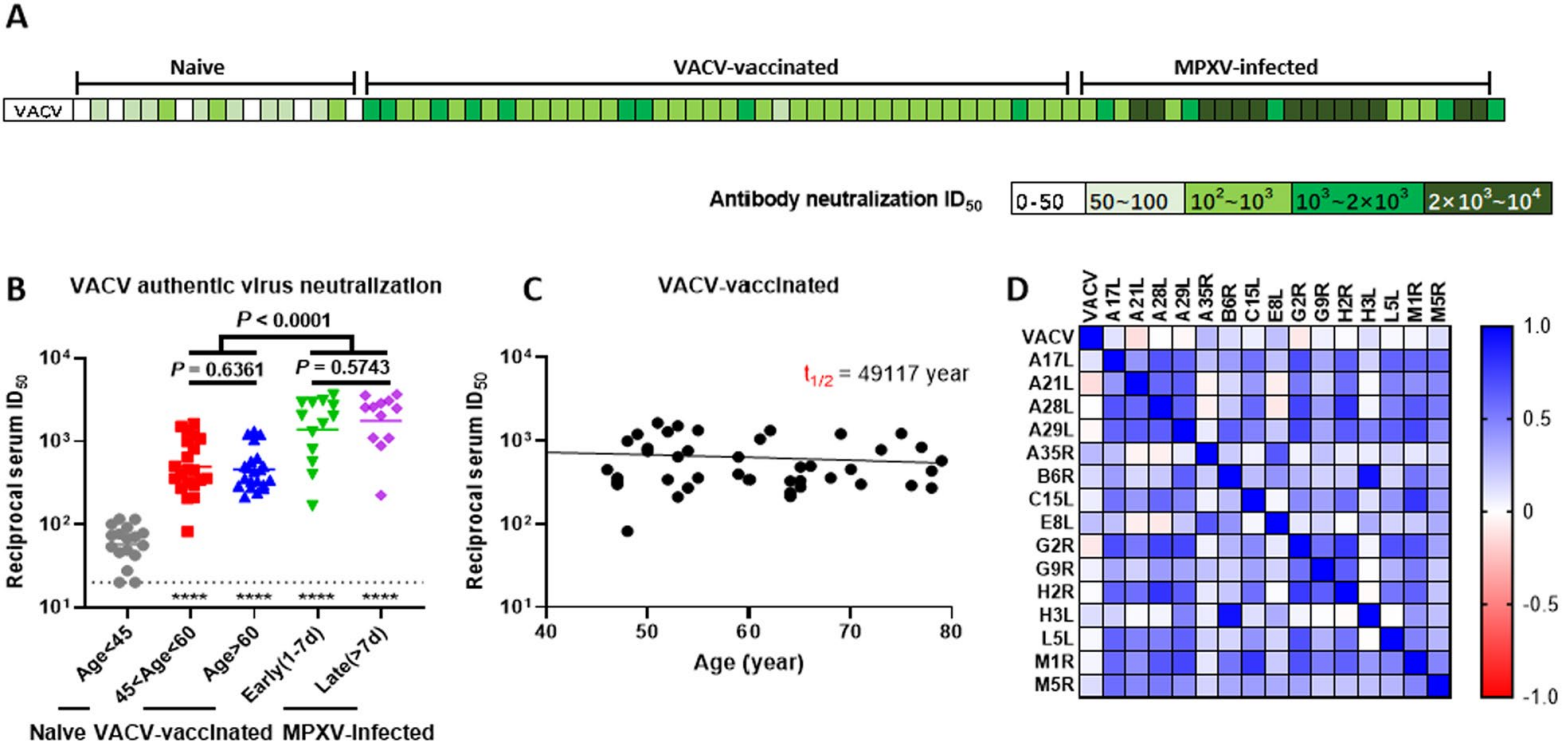
Correlation analysis of antibody binding to MPXV antigens and neutralization against VACV. **A** ID_50_ of the participants’ serum samples against VACV determined via neutralization tests. **B** Differential VACV neutralization ability between naive (Age < 45), VACV-vaccinated (45 < age < 60 or age > 60) and MPXV-infected (1 ~ 7 or > 7 d.p.i) individuals (compared with naive individuals, *P < 0.05). **C** Decay trend of neutralization activity in VACV-vaccinated individuals (t_1/2_ = 49,117 year). **D** Pearson correlation matrix of antibody binding to MPXV antigens and the VACV neutralization capacity. A two-tailed correlation was performed using all the ELISA data for the MPXV antigens and the VACV neutralization results. Only significant correlations are shown in dark blue (positive) or red (negative), with blank cells indicating nonsignificant correlations. d.p.i, Days post infection; ID_50,_ 50% inhibitory dilutions; MPXV, Monkeypox virus; t_1/2,_ Decay half-life; VACV, Vaccinia virus

The infection rate was evaluated by measuring GFP signals using high content analysis system, with a greater number of GFP signals indicating a higher level of VACV infection (Figure S4). Serum samples collected from MPXV-infected or VACV-vaccinated individuals efficiently suppressed of VACV infection, and samples in these groups could neutralize VACV at serum dilutions ranging from 1: 10^2^ to 1: 10^4^. In contrast, serum from naive individuals exhibited only very low levels of neutralization activity against VACV infection (Fig. 4A). Notably, serum from VACV-vaccinated individuals still demonstrated neutralization against VACV-WR strain even after more than 40 years post-vaccination. Among the VACV-vaccinated group, individuals over 60 years old showed no significant difference in neutralization activity compared to those aged from 45 to 60 years (*P* = 0.6361, Fig. 4B). When we analyzed the neutralization decay trend for all the VACV-vaccinated individuals based on their age, we found that VACV vaccination produced stable serum neutralization with an antibody decay half-life (t_1/2_) of 49,117 years (Fig. 4C). These results indicated that the serum sample neutralization capacity remained consistent over time in VACV-vaccinated individuals. However, compared to the VACV vaccination group, individuals with recent MPXV-infection showed overall significantly higher neutralization activity (*P* < 0.0001, Fig. 4B), and a subset of MPXV-infected individuals exhibited strong serum neutralizing antibody responses, with neutralization ID_50_ values greater than 2000 (Fig. 4A, B). No significant factor that influenced the VACV serum neutralization capacity was identified via binary regression analysis (*P* > 0.11, Tables S2 and S3).

We employed Pearson correlation to determine the relationships among antibody responses to various MPXV antigens and their relevance to VACV neutralization capacity (Fig. 4D). The heatmap revealed that the ability of serum samples to neutralize VACV correlated most positively with antibody binding to A35R and E8L. Additionally, A29L was associated with A28L, A21L and A17L. Significant correlations were also identified among A29L, A28L, A21L, A17L, G2R, and H2R, which are core members of the EFC (*P* < 0.0001). A35R was more closely correlated with E8L, and increased antibody response to both were observed in late infection phase of MPXV-infected individuals. The strongest correlation between B6R and H3L (the lowest* P* = 1.097 × 10^–38^ and the highest Pearson* r* = 0.934) suggests simultaneous production of corresponding antibodies. The strong correlations observed between proteins with similar membrane location indicate that these proteins maintain their original conformation when inducing antibody responses. Conversely, there was a negative correlation of antibody responses when compared A35R, E8L to A21L, A28L. The VACV neutralization capacity was also negatively correlated with antibody binding to A21L and G2R.

## Discussion

### Antigens A35R, B6R, H3L and E8L are identified as highly immunogenic antigens that elicit a robust antibody response in MPXV-infected individuals

MPXV, along with other OPXV, exhibits a notably intricate viral life cycle, encoding over 200 viral proteins [37]. Our research has shown that the antibody responses generated by the smallpox vaccine (VACV-TT) and prior MPXV infections (cases located in Nanjing, 2023–2024) were both high for the antigens A35R, B6R, H3L and E8L, in contrast to naive samples from individuals who had neither received VACV vaccination nor experienced MPXV infection. The strong antibody response to these antigens following MPXV infection aligns with previous studies revealing the importance of targeting A35R, B6R, H3L or E8L [16, 38–40]. Our laboratory is planning to investigate human monoclonal antibodies specific to these antigens for their potential applications in the prevention and treatment of mpox. Other antigens did not show a very strong antibody response among all groups, which may be attributed to their functions and lower conservation than their VACV counterparts. Consequently, the immune mechanisms underlying this phenomenon, such as immune selection, warrant further investigation in subsequent studies. We conducted a thorough analysis of the breadth and coverage of antibodies induced by VACV-vaccination and MPXV-infection. MPXV-infection led to stronger neutralization of VACV and binding antibody responses to A35R, H3L and E8L. The neutralizing ability against VACV was significantly stronger in serum samples obtained from individuals infected with MPXV than in those from individuals vaccinated with VACV. Pearson correlation analysis indicated strong associations among the surface antigens of MPXV. Specifically, the envelope protein A35R of EEV demonstrated a close relationship with the attachment protein E8L, while the protein B6R exhibited a strong correlation with H3L. Notably, antibody responses to both A35R and E8L showed substantial increases in individuals infected with MPXV during the late phase of infection. These four antigens, which display considerable immunogenicity, are interconnected.

### The presence of cross-reactive and enduring antibody responses to surface proteins has been observed in individuals who have received Chinese smallpox vaccinations

In our study, serum samples from individuals vaccinated with VACV demonstrated increased binding affinity to the antigens A21L, A28L, A29L, G2R, and H2R. The variations in binding antibody responses to distinct MPXV antigens were found to be correlated with the degree of their amino acid sequence similarity among OPXVs (Pearson correlation coefficient *r* = 0.6847, *P* < 0.05). Specifically, the higher levels of amino acid sequence conservation observed in the EFC A21L, G2R, and H2R, as well as the attachment protein A28L, may account for the enhanced antibody responses elicited by VACV vaccination. The minimal reactions observed to certain antigens in the naive group could be attributed to incidental exposure to OPXVs. Additionally, individuals aged over 60 years old exhibited antibody responses that were comparable to those of younger age cohorts, highlighting the long-lasting efficacy and importance of VACV vaccination. Consistent with findings from previous studies, the diminished antibody responses and neutralization capacity noted in our study may be a consequence of MPXV infection occurring within the last 6 months, in contrast to the vaccination that took place over 40 years ago [41]. Consequently, the administration of booster doses and the consideration of modified smallpox vaccinations should be prioritized to elicit more robust antibody responses [42]. Furthermore, notable correlations were identified among the antigens A21L, A28L, A29L, G2R, and H2R, which exhibited enhanced antibody responses induced by VACV vaccination. Therefore, it is both logical and strategic to explore the development of cocktail therapies or poly-specific antibodies informed by these correlations.

### The conservation of MPXV proteins through representative strains facilitates the utilization of highly immunogenic antigens in the development of vaccines and antibody-based therapeutic strategies

The conservation and variation in OPXV are crucial mechanisms that help us understand certain phenomena related to mpox. Notably, epidemiological evidence has confirmed the emergence of the new C.1.1 lineage in China due to mutations. The mutation rate of MPXV appears to have risen in recent years, showing distinct continental characteristics and diverging into two clades. The importation events within the clade IIb C.1 lineage in our phylogenetic analysis reveal a clear genomic connection to the Asian region from 2023 to 2024. This highlights the importance of addressing the clade IIb C.1 and clade Ib lineages in China. Consequently, we selected 9 representative strains from clade Ia to clade IIb C.1.1. For these strains, the amino acid sequence similarity between the 15 MPXV antigens and their corresponding OPXV antigens exceeds 93.75%. On 3 January 2025, China notified WHO of the first cases of mpox due to clade Ib MPXV detected in the country so far[9]. The ongoing mutations in MPXV lead to micro-evolution in our targeted antigens, ensuring that the highly immunogenic antigens remain valuable for vaccine and antibody development strategies in response to outbreaks of clade IIb C.1.1 MPXV and clade Ib MPXV.

### Limitations exist in our study

There are limitations in our study that need to be addressed. First, the number of MPXV-infected individuals involved in our cohort was limited. Second, no clade Ib cases were identified in China when we established this cohort, so our study focused on clade IIb cases. Third, studies of MPXV require BSL-3 containment due to biosafety level constraints, so we just utilized another OPXV (VACV) to perform the neutralization experiments.

## Conclusions

This study demonstrates that it is plausible to develop immunogen strategies and MPXV-neutralizing antibodies targeting core immunogenic antigens A35R, B6R, H3L and E8L. Given the cross-reactive antibody responses and the sustained, comprehensive protection from VACV-vaccination, administering booster doses of the smallpox vaccine in high-risk populations is rational approach for rapid outbreak prevention and control.

## Supplementary Information

Supplementary Material 1: Figure S1: Blood test results of MPXV-infected individuals. Most participants had HIV viral loads below 100 and CD4 + counts greater than 500, consistent with their prior antiretroviral therapy progression. The majority of MPXV-infected individuals tested negative for MPXV within ten days after undergoing two tests for viral load. HIV, Human immunodeficiency virus; MPXV, Monkeypox virus.

Supplementary Material 2: Figure S2: Antibody binding curves to 15 MPXV antigens from serial dilutions of serum samples from (A) naive individuals, (B) VACV-vaccinated individuals, and (C) MPXV-infected individuals. MPXV, Monkeypox virus; VACV, Vaccinia virus.

Supplementary Material 3: Figure S3: Amino acid sequence comparisons of 15 MPXV antigens with their homologs in VACV, CPXV, and smallpox virus SPXV. The GenBank protein sequence numbers for each comparison are listed on the left side. From top to bottom, the MPXV genome sequences are: hMpxV/China/GZ8H-01/2023, hMpxV/human/CHN/GDCDC_SZ_M23254/2023, MPXV-M5312_HM12_Rivers, MPXV_USA_2022_FL001, Monkeypox virus isolate VSP188, Monkeypox virus isolate CHVir44025_Sep2023, Zaire-96-I-16, Monkeypox_virus_isolate_24MPX0223C, MPXV/human/Japan/Tokyo/NCGM240303/2024, VACV_TT11_001, VACV_WR, Cowpox_Brighton_Red and Variola_virus_strain_China_Horn_1948.MPXV, Monkeypox virus; OPXV, Orthopoxvirus; SPXV, Smallpox virus; TT, Tiantan; VACV, Vaccinia virus; WR, Western Reserve.

Supplementary Material 4: Figure S4: VACV neutralization by participants’ serum samples. (A) Representative image of the VACV neutralization test captured by high content analysis system. (B) Neutralization curves of serum samples from naive individuals, VACV-vaccinated individuals, and MPXV-infected individuals. MPXV, Monkeypox virus; VACV, Vaccinia virus

Supplementary Material 5: Table S1. Basic information of complete genome sequences in phylogenetic tree. Table S2. Factors associated with the seropositivity of antibody and VACV neutralization capacity in all groups (sex, MPXV infection, VACV vaccinated and age) Table S3. Factors associated with the seropositivity of antibody and VACV neutralization capacity in MPXV-infected individuals (MXPV CT value, CD4 + count, CD4 + /CD8 + and HIV viral load). Table S4. MPXV antigen amino acid similarity within OPXVs. CI,confidence interval; EC_50_, Median effect concentration; HIV, Human immunodeficiency virus; ID_50_, 50% inhibitory dilutions; MPXV, Monkeypox virus; OPXV, Orthopoxvirus; SPXV, Smallpox virus; TT, Tiantan; VACV, Vaccinia virus; WR, Western Reserve.

## Data Availability

All data generated or analyzed during this study are included in this published article and Supplemental Material (xx.docx). Fig. S1 to S4 & Table S1 to S4.

## Abbreviations

CI: Confidence interval
CPXV: Cowpox virus
d.p.i: Days post infection
DRC: Democratic Republic of the Congo
t_1/2_: Decay half-life
EC_50_: Median effect concentration
EEV: Extracellular enveloped virion
EFC: Entry fusion complex
ELISA: Enzyme-linked immunosorbent assay
ID_50_: 50% Inhibitory dilutions
HIV: Human immunodeficiency virus
IQR: Interquartile range
MV: Mature virion
IMV: Intracellular mature virion
Mpox: Monkeypox
MPXV: Monkeypox virus
OD_450_: Optical Density 450
*OR*: Odds ratio
OPXV: Orthopoxvirus
SPXV: Smallpox virus
TT: Tiantan
VACV: Vaccinia virus
WHO: World Health Organization
WR: Western Reserve
