# Supplementary figures and images for: Immunogenicity of monkeypox virus surface proteins and cross-reactive antibody responses in vaccinated and infected individuals: implications for vaccine and therapeutic development
Source: Infect Dis Poverty. 2025 Feb 25;14:12. doi: 10.1186/s40249-025-01280-1 (PMC11852519; doi:10.1186/s40249-025-01280-1)

## Slide 1
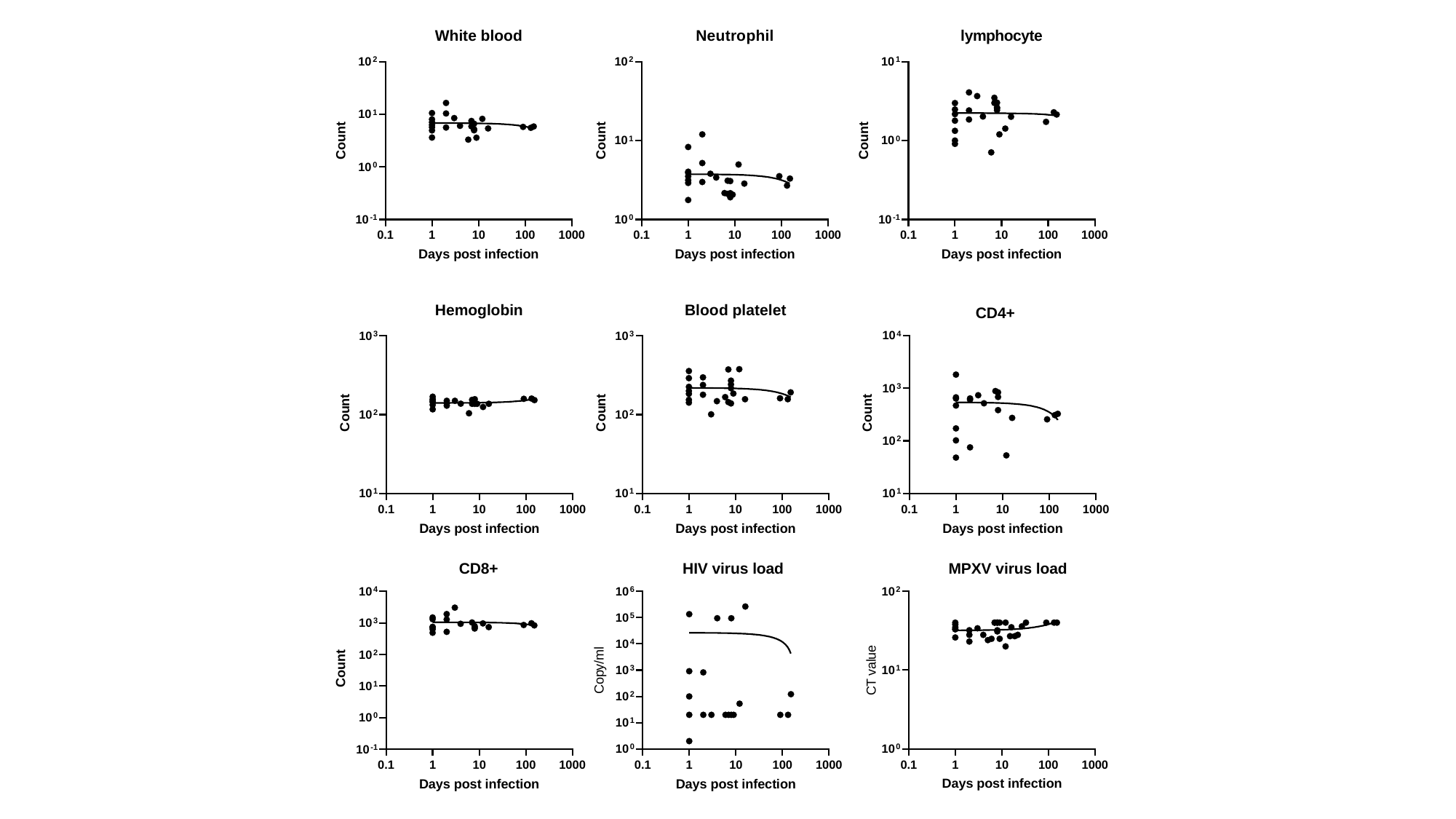

Supplement: Supplementary file 1 — Supplementary Material 1: Figure S1: Blood test results of MPXV-infected individuals. Most participants had HIV viral loads below 100 and CD4 + counts greater than 500, consistent with their prior antiretroviral therapy progression. The majority of MPXV-infected individuals tested negative for MPXV within ten days after undergoing two tests for viral load. HIV, Human immunodeficiency virus; MPXV, Monkeypox virus. [file 40249_2025_1280_MOESM1_ESM.pptx]

## Slide 1
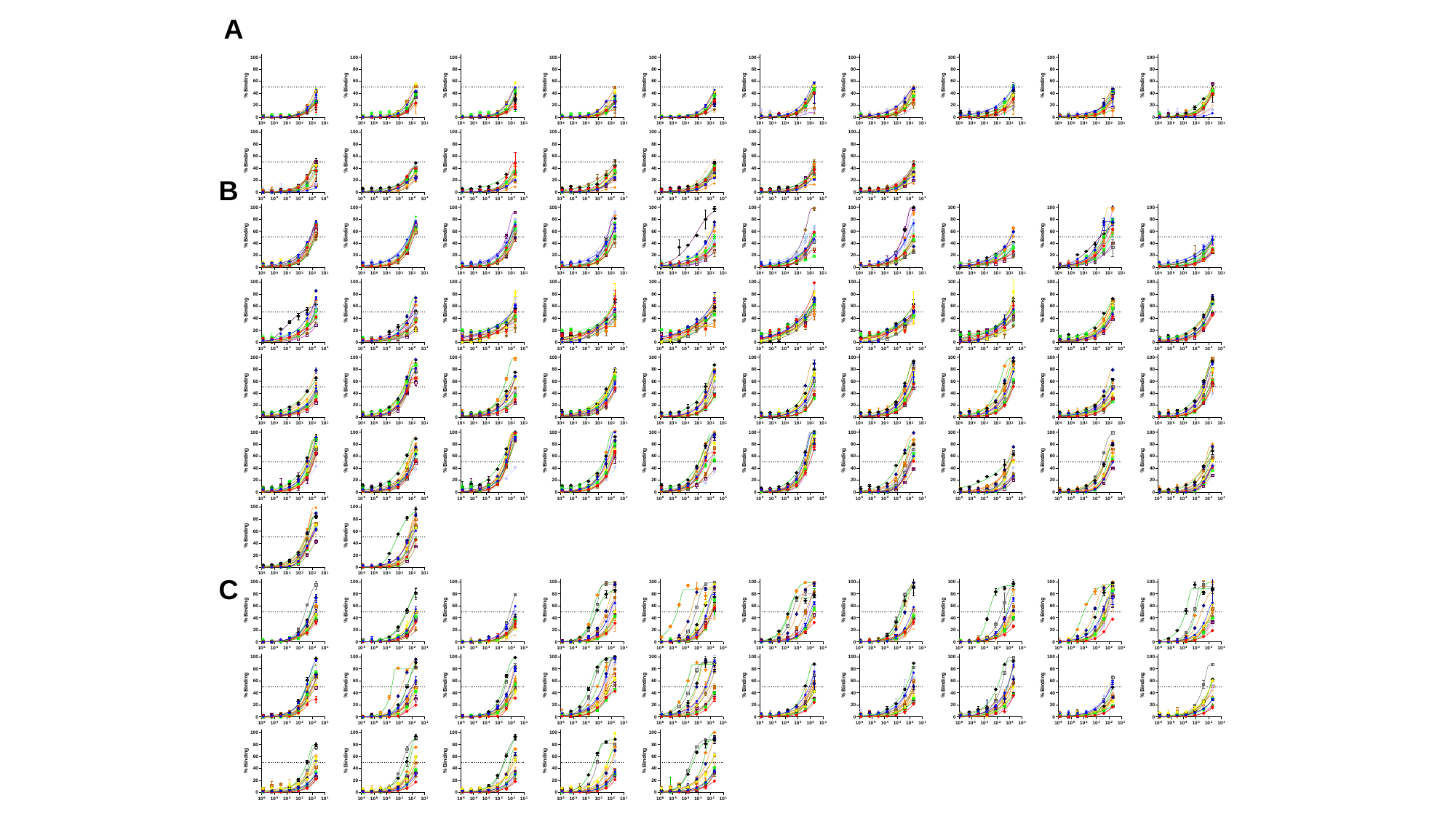

Supplement: Supplementary file 2 — Supplementary Material 2: Figure S2: Antibody binding curves to 15 MPXV antigens from serial dilutions of serum samples from (A) naive individuals, (B) VACV-vaccinated individuals, and (C) MPXV-infected individuals. MPXV, Monkeypox virus; VACV, Vaccinia virus. [file 40249_2025_1280_MOESM2_ESM.pptx]

## Slide 1
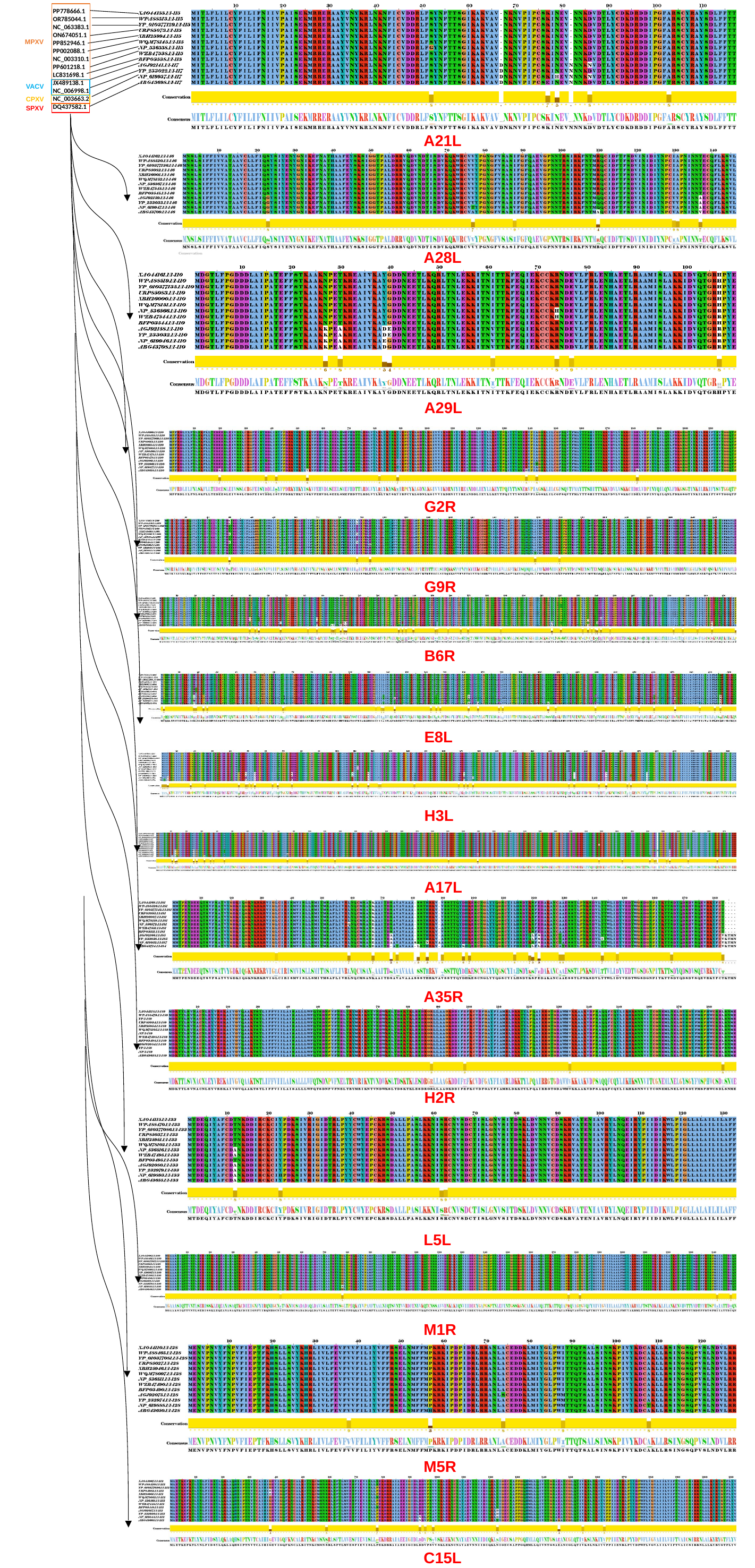

A21L
A28L
A29L
G2R
G9R
B6R
E8L
H3L
A17L
A35R
H2R
L5L
M1R
M5R
C15L
PP778666.1
OR785044.1
NC_063383.1
ON674051.1
PP852946.1
PP002088.1
NC_003310.1
PP601218.1
LC831698.1
JX489138.1 NC_006998.1 NC_003663.2 DQ437582.1
MPXV
VACV
CPXV
SPXV

Supplement: Supplementary file 3 — Supplementary Material 3: Figure S3: Amino acid sequence comparisons of 15 MPXV antigens with their homologs in VACV, CPXV, and smallpox virus SPXV. The GenBank protein sequence numbers for each comparison are listed on the left side. From top to bottom, the MPXV genome sequences are: hMpxV/China/GZ8H-01/2023, hMpxV/human/CHN/GDCDC_SZ_M23254/2023, MPXV-M5312_HM12_Rivers, MPXV_USA_2022_FL001, Monkeypox virus isolate VSP188, Monkeypox virus isolate CHVir44025_Sep2023, Zaire-96-I-16, Monkeypox_virus_isolate_24MPX0223C, MPXV/human/Japan/Tokyo/NCGM240303/2024, VACV_TT11_001, VACV_WR, Cowpox_Brighton_Red and Variola_virus_strain_China_Horn_1948.MPXV, Monkeypox virus; OPXV, Orthopoxvirus; SPXV, Smallpox virus; TT, Tiantan; VACV, Vaccinia virus; WR, Western Reserve. [file 40249_2025_1280_MOESM3_ESM.pptx]

## Slide 1
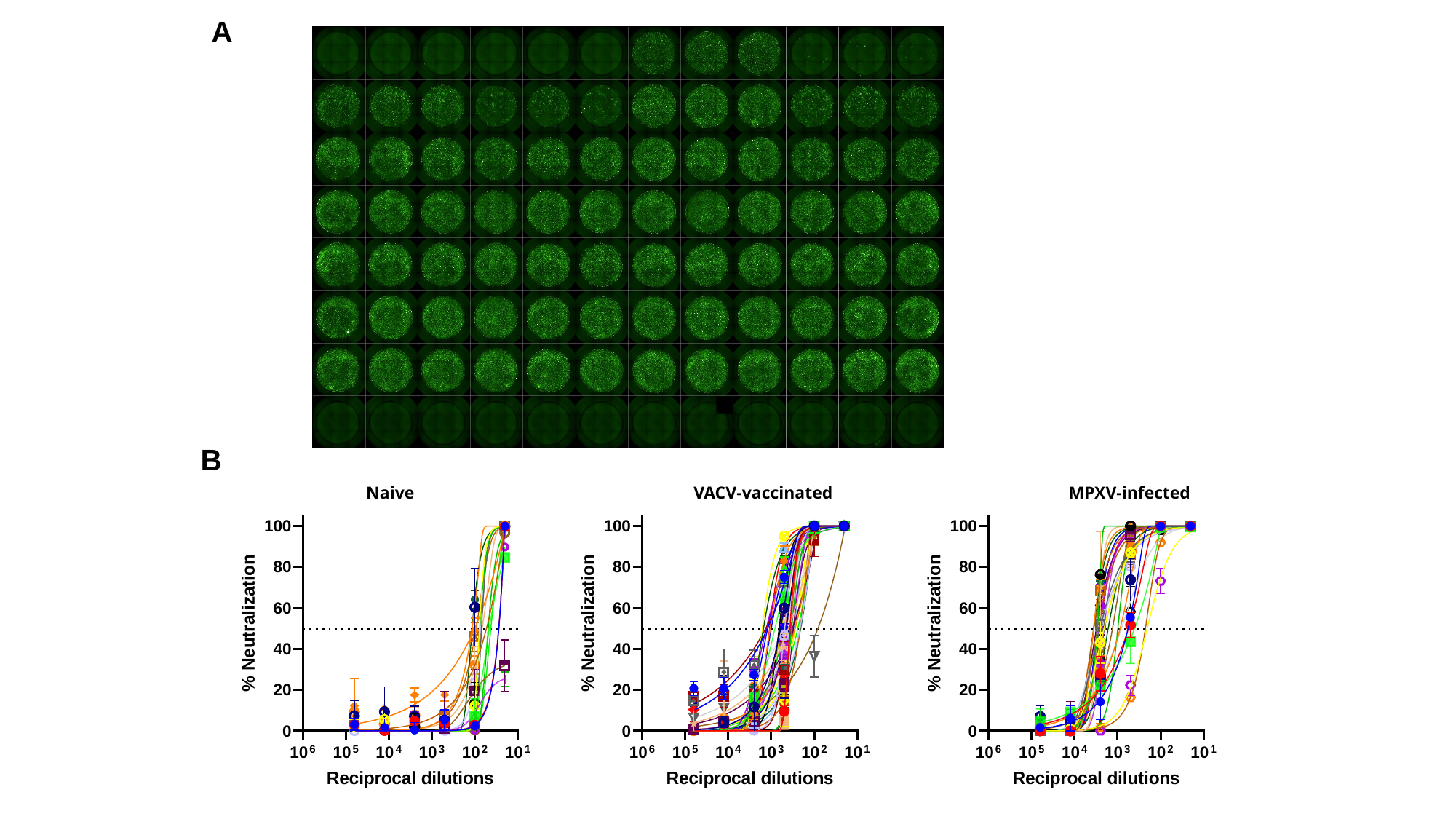

Naive
VACV-vaccinated
MPXV-infected

Supplement: Supplementary file 4 — Supplementary Material 4: Figure S4: VACV neutralization by participants’ serum samples. (A) Representative image of the VACV neutralization test captured by high content analysis system. (B) Neutralization curves of serum samples from naive individuals, VACV-vaccinated individuals, and MPXV-infected individuals. MPXV, Monkeypox virus; VACV, Vaccinia virus [file 40249_2025_1280_MOESM4_ESM.pptx]
